# Randomized controlled trial of ultra-protective vs. protective ventilation strategy in veno-arterial extracorporeal membrane oxygenation patients with refractory cardiogenic shock: a study protocol for the ultra-ECMO trial

**DOI:** 10.3389/fcvm.2023.1092653

**Published:** 2023-05-05

**Authors:** Wei Li, Chen Chen, Deliang Hu, Feng Sun, Gang Zhang, Zhongman Zhang, Yanbin Dong, Jinru Lv, Yong Mei, Xufeng Chen

**Affiliations:** Department of Emergency Medicine, The First Affiliated Hospital of Nanjing Medical University, Nanjing, China

**Keywords:** cardiogenic pulmonary edema (CPE), ventilator-free days, protective ventilation, ultra-protective ventilation, veno-arterial extracorporeal membrane oxygenation (VA ECMO)

## Abstract

**Background:**

A protective or ultra-protective tidal volume strategy is widely applied to patients with acute respiratory distress syndrome (ARDS). The use of very low tidal volume has the potential to further redece ventilation-induced lung injury (VILI) comparde with a “normal” lung protective management. Plus, cardiogenic pulmonary edema (CPE) caused by hydrostatic mechanisms in patients with cardiogenic shock has similar respiratory mechanics to those found in patients with ARDS. And no consensus exists on mechanical ventilation parameter settings in patients with VA-ECMO. The study aimed to investigate the impact of an ultra-protective tidal volume strategy on the 28-day ventilator-free day (VFD) number in VA-ECMO–supported patients with refractory cardiogenic shock, including cardiac arrest.

**Methods:**

The Ultra-ECMO trial is a randomized controlled, open-label, single-center prospective superiority trial. At the onset of ECMO initiation, we will divide patients randomly into an intervention group and a control group in a 1:1 ratio. The control group will adopt protective ventilation settings [initial tidal volume: 6 ml/kg of predicted body weight (PBW)] for ventilation, and the intervention group will adopt ultra-protective ventilation settings (initial tidal volume: 4 ml/kg of PBW) for ventilation. The procedure is expected to last 72 h, after which the ventilator settings will be at the intensivists' discretion. The primary outcome is the VFD number at 28 days after inclusion. The secondary outcomes will include respiratory mechanics; analgesic/sedation dosage; lung ultrasound score; interleukin-6, interleukin-8, and monocyte chemotactic protein-1 levels in broncho-alveolar lavage fluid at the moment of enrollment (T0), 24, 48, and 72 h (T1, T2, and T3, respectively) after ECMO initiation; total time (in days) required for ECMO weaning; length of stay in the intensive care unit; total cost of hospitalization; amounts of resuscitative fluids; and in-hospital mortality.

**Discussion:**

VA-ECMO–treated patients without ARDS possess abnormal lung function. CPE, thoracic compliance reduction, and poor pulmonary blood perfusion are frequently present, and these patients can more easily progress to ARDS. It seems that targeting the protective tidal volume can lower adverse outcome incidence rates, even in patients without ARDS. This trial seeks to answer the question of whether adopting an ultra-protective tidal volume strategy can lead to superior primary and secondary outcomes compared to adopting a protective tidal volume strategy in patients treated by VA-ECMO. The Ultra-ECMO trial will provide an innovative mechanical ventilation strategy for VA-ECMO–supported patients for improving treatment outcomes at biological and potentially clinical levels.

**Clinical Trial Registration:**

ChiCTR2200067118.

## 1. Introduction

Refractory cardiogenic shock (RCS), including cardiac arrest (CA), is characterized by severe circulation failure accompanied by cardiogenic pulmonary edema (CPE) and often necessitates invasive mechanical ventilation (IMV) (1). In the most severe cases of RCS, veno-arterial extracorporeal membrane oxygenation (VA-ECMO) is a viable therapy used to maintain a certain amount of cardiac output (2). Although advances in VA-ECMO technology have significantly improved the clinical outcomes of patients suffering from RCS or CA in the past decades (3), there is a paucity of high-grade scientific evidence suggesting the optimal ventilator settings during VA-ECMO treatment. The Extracorporeal Life Support Organization has proposed a ventilation strategy with a positive end-expiratory pressure (PEEP) of ≥10 cmH_2_O and low minute ventilation without offering further specific details (4). In a recent observational study of 256 out-of-hospital CA (OHCA) patients, a decrease of 1 ml/kg PBW in the tidal volume (VT) during the first 48 h of intensive care unit (ICU) admission after OHCA was associated with a 61% increase in adjusted favorable neurocognitive outcomes, suggesting that a lower VT in the early phase of ICU admission may influence patient prognosis (5). However, a similar relationship has not been found among in-hospital CA patients (6).

To date, studies have advocated for the application of an ultra-protective VT strategy in patients with ARDS being treated by veno-veno ECMO (VV-ECMO) (7–9). Of note, CPE caused by hydrostatic mechanisms in patients with cardiogenic shock may have similar respiratory mechanics to those seen in patients with ARDS, although the pathophysiology is different. Similarly, IMV during VA-ECMO with ultra-protective VT may attenuate ventilator-induced lung injury due to the increased strain/stress and mechanical power transmitted by the ventilator. Thus, we hypothesized that adopting an ultra-protective VT strategy during the early phase of VA-ECMO may be a credible option to facilitate IMV weaning and even reduce in-hospital mortality. The results of this randomized controlled trial may subsequently add a novel and viable treatment solution to routine clinical practice involving VA-ECMO patients.

## 2. Methods and analysis

### 2.1. Trial design

Ultra-ECMO trial is a single-center, prospective clinical trial that will enroll 2 parallel groups of patients in a 1:1 ratio. It is designed to evaluate the 28-day number of ventilator-free days (VFDs) following an early, short-course application (72 h) of an ultra-protective VT vs. protective VT strategy in patients on VA-ECMO (Table 1) (1). Table 2 overviews the trial registration information. This trial protocol conforms to the Consolidated Standards of Reporting Trials (CONSORT) statements (10, 11) and has obtained the approval of the Research Ethics Committee at the First Affiliated Hospital of Nanjing Medical University. The legal guardians of eligible patients will sign the informed consent following the Declaration of Helsinki of 1964 (revised in 2013) (12).

**Table 1:** Indications and contraindications for VA-ECMO.

Indications for VA-ECMO
1. Extracorporeal cardiopulmonary resuscitation (ECPR)
2. Acute myocardial infarction
3. Acute myocarditis
4. Progression of cardiomyopathy, ischemic or nonischemic
5. Acute RV failure due to pulmonary embolism
6. Progression of RV failure due to pulmonary disease
7. Progression of congenital heart disease
8. Primary graft failure and acute allograft rejection after heart transplantation
9. Overdose of cardiotoxic drugs
10. Septic cardiomyopathy
11. Trauma
12. Refractory ventricular tachycardia
13. RV failure during LVAD support
14. Failure to wean off cardiopulmonary bypass
Contraindications for VA-ECMO
1. Severe irreversible noncardiac organ failure limiting survival (e.g., severe anoxic brain injury or metastatic cancer)
2. Irreversible cardiac failure if transplantation or long term LVAD are not considered
3. Aortic dissection
4. Severe coagulopathy or contraindication to anticoagulation, including advanced liver disease
5. Limited vascular access (severe peripheral arterial disease, extreme obesity, amputated limbs, among others)

LVAD, left ventricular assist device; VA-ECMO, veno-arterial extracorporeal membrane oxygenation; RV, right ventricular.

**Table 2:** The trial registration for the ultra-ECMO trial.

Data category	Information
Primary registry and trial identifying number	ChiCTR2200067118
Trial protocol version	Version 1
Source of monetary or material support	The National Natural Science Foundation of China
Primary sponsor	The National Natural Science Foundation of China
Secondary sponsor	N/A
Contact for public queries	XFC, cxfyx@njmu.edu.cn YM, meiyong@jsph.org.cn JRL, lvjinru@jsph.org.cn
Public title	Randomized Controlled Trial of Ultra-Protective vs. Protective mechanical ventilation strategy in veno-arterial Extracorporeal membrane oxygenation patients with Refractory cardiogenic shock: study protocol for the Ultra-ECMO trial
Country	China
Study type	Type: Randomized controlled, parallel group, clinical trial Group allocation: Simple randomization Masking: data analyst, statistician blinded
Date of first enrollment	Not yet started
Target sample size	614 in total
Primary outcome(s)	The 28-day ventilator-free day
Key secondary outcome(s)	All-cause in-hospital mortality, level of nflammatory factors in BAL, time to VA-ECMO weaning, length of ICU stay,respiratory mechanics parameters, lung ultrasound score, total analgesic/sedation dosage, total cost for hospitalization, amounts of fluid resuscitation

### 2.2. Trial objectives

This trial has the main objective of assessing the potential benefit of an ultra-protective VT strategy relative to a protective VT strategy on the 28-day VFD number (13) (Table 3).

**Table 3:** Ready for invasive mechanical ventialtion weaning checklist.

Step 1: Patient's clinical condition is appropriate for attempting to decrease the level of respiratory support, e.g.: without severe shock or multi-organ failure
Step 2: Patient is awake enough to at least protect his or her airway, cooperative enough not to be at significant risk for dislodgement of cannulas or other important catheters or devices, and not requiring heavy or frequent sedation
Step 3: Secretions are manageable without an artificial airway
Step 4: The patient should have an acceptable arterial blood gas on minimal ventilator settings (e.g.: FiO2 0.4, PEEP 5):Goal PaO2 > 80 mmHg, pH > 7.35 with minute ventilation < 10 L/min
Step 5: Weaning of mechanical ventilation

FiO2, fraction of inspired oxygen; PEEP, positive end-expiratory pressure; PaO2, partial pressure of arterial oxygen.

**Figure 1:**
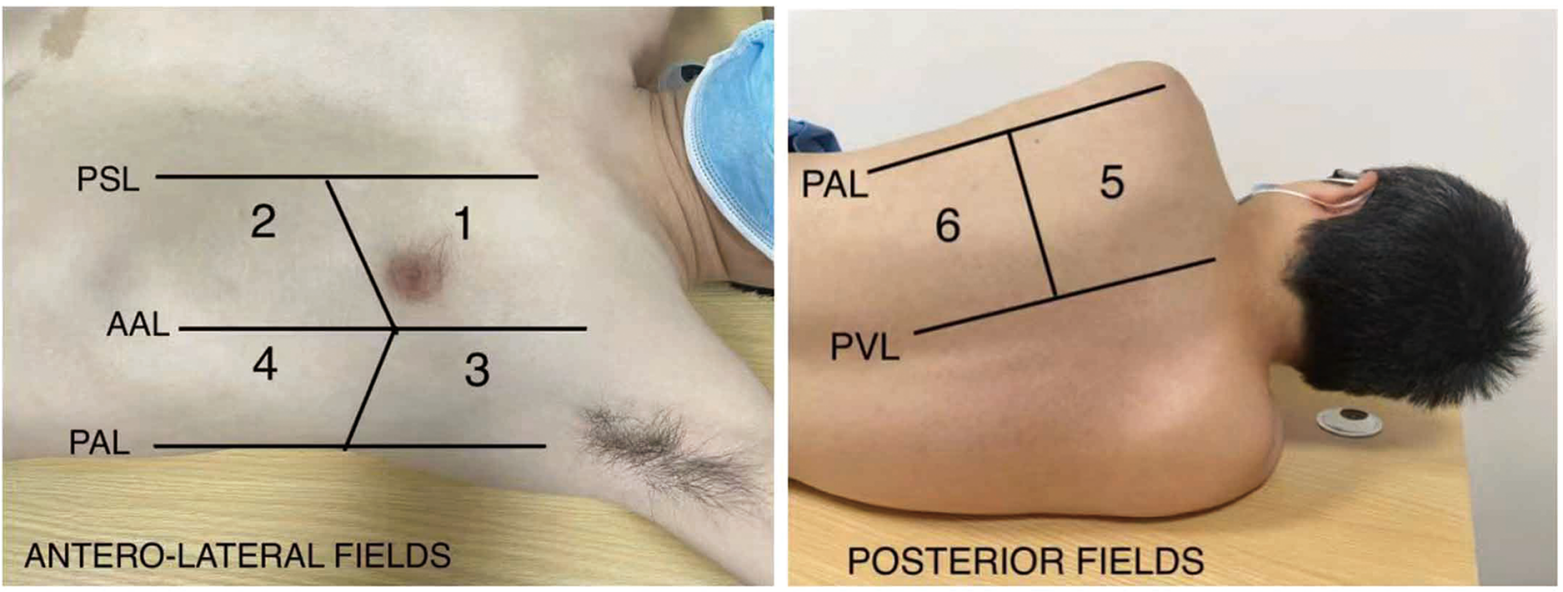
Lung ultrasound regions. PSL, parsternal line; AAL, anterior axillary line; PAL, posterior axillary line; PVL, paravertebral line.

Meanwhile, the secondary objectives of this study include the following:

1. To examine whether ultra-protective VT can decrease the all-cause in-hospital mortality rate
2. To examine whether ultra-protective VT can reduce the time for successful VA-ECMO weaning (Table 4)
3. To examine whether ultra-protective VT is associated with a shorter ICU length of stay
4. To examine whether ultra-protective VT is associated with a reduction in interleukin-6 (IL-6), interleukin-8 (IL-8), and monocyte chemotactic protein-1 (MCP-1) levels in broncho-alveolar lavage fluid (BALF)
5. To examine whether ultra-protective VT affects respiratory parameters (e.g., total PEEP, plateau pressure [P_plat_], driving pressure [DP], airway resistance [R], static compliance [C_stat_], mean airway pressure [P_mean_], esophageal pressure [P_es_], transpulmonary pressure [P_tp_], and mechanical power [MP]), based on a daily assessment from inclusion to closeout (14)
6. To examine whether the ultra-protective VT strategy decreases the lung ultrasound score (LUS) (Figure 1 and Table 5) when assessed daily from inclusion to closeout (15, 16)
7. To examine whether ultra-protective VT can lead to an increased analgesic/sedation dosage during the intervention period after inclusion
8. To examine whether ultra-protective VT predicts a decrease in the total hospitalization cost
9. To examine whether ultra-protective VT is associated with a reduced demand for fluid resuscitation

**Table 4:** Ready for VA-ECMO weaning checklist.

VA ECMO weaning should be considered when patients exhibit stable hemodynamics with reduced ECMO flow, even if n low doses vasoactive or inotropic Support is mandatory
1. MAP > 60 mmHg
2. LVOT VTI > 0.12 m/s
3. Tissue Doppler lateral mitral annulus peak systolic velocity ≥6 cm/sec
4. CVP ≤ 10 mmHg, and LV ejection fraction ≥25%–30% on low doses vasoactive, inotropic support.
5. All the above mentioned conditions are fulfilled indicating the patient is readey for VA-ECMO weaning

VTI, velocity time integral; CVP, central venous pressure; LVOT, left ventricular outflow tract; MAP, mean arterial pressure.

**Table 5:** Calculaiton of lung ultrasound score (LUS).

	Aeration	Lung ultrasound appearance
Score 0	normal	A lines-max 2 B lines
Score 1	moderate loss	B1 lines (≥3 well-spaced B lines with horizontal spacing between adjacent B lines ≤7 mm)
Score 2	severe loss	B2 lines (multiple B lines fused with horizontal spacing between adjacent B lines ≤3 mm)
Score 3	complete loss	echoic lung tissue, accompanied by dynamic air bronchogram
Total score		Global LUS was calculated by summing the highest score of each region (0–36)

### 2.3. Trial setting

We will conduct the Ultra-ECMO trial in the Department of Emergency Medicine at the First Affiliated Hospital of Nanjing Medical University, which is capable of initiating ECMO therapy for nearly 100 critically ill patients annually.

### 2.4. Inclusion and exclusion criteria

Patients ≥18 years of age receiving VA-ECMO therapy, such as extracorporeal cardiopulmonary resuscitation, will be eligible for inclusion in the Ultra-ECMO trial. Additional key inclusion criteria include (1) IMV onset of >72 h, (2) no pregnancy, (3) ECMO duration of >72 h, (4) RCS or CA with a cardiac origin, (5) no enrollment in another clinical trial, (6) a consent form signed by the guardian has been provided, and (7) a description of CPE on high-resolution chest computed tomography images has been given by two radiologists with ≥5 years of diagnostic experience who are unaware of the clinical diagnosis.

Meanwhile, the exclusion criteria for patient enrollment are as follows: (1) trauma; (2) written informed consent has not been provided by close relatives; (3) the patient has contraindications for fiberoptic bronchoscopy examination; (4) lung ultrasound images are blurry; (5) pneumothorax or massive pleural effusion; (6) ECMO is being used as a bridge for heart transplantation; and (7) the patient developed adverse events that complicate weaning from IMV (e.g., intracranial hemorrhage, thromboembolism, severe infection).

### 2.5. Recruitment, randomization, and blinding

The Ultra-ECMO trial will include patients who meet the abovementioned eligibility criteria. Formal information about the trial and an official copy of the informed consent form will be sent to their guardians. We will assign participants to an intervention arm or a control arm in a random manner, with those in the former group receiving ultra-protective VT and those in the latter receiving 72 h of protective VT. Guardians will also be informed that they are allowed to drop out of the trial at any time.

The randomization list will be computer-generated according to a simple randomization principle using a ratio of 1:1 by the Clinical Research Board of the School of Public Health of Nanjing Medical University. Participants will be assigned to each group by an independent trial assistant after verification of patient eligibility. Once group allocation has been completed, local investigators will register the record, then reveal the group allocation to care providers, who will deliver the intervention.

Trial assistants responsible for group allocation will be blinded to both groups of patients, but blinding of care providers is impracticable as ventilatory settings and respiratory mechanics measurements are part of routine daily clinical practice. The outcome assessors, data analysts and statisticians will not know the group allocation.

### 2.6. Interventions

At the onset of ECMO initiation, all participants will be screened by therapists. Once the patient meets the inclusion criteria and is finally enrolled, the therapists will provide the selected intervention. We will assign patients in the intervention group to receive ultra-protective VT after randomization (initial settings: VT, 4 ml/kg of PBW; PEEP, 10 cmH_2_O; respiratory rate, 10 min^−1^; Vt may be decreased stepwise by 0.5 ml/kg PBW if the plateau pressure is >30 cmH_2_O) (17).

Patients in the control group will be managed using a protective VT strategy after randomization (initial settings: VT, 6 ml/kg of PBW; PEEP, 10 cmH_2_O; respiratory rate, 10 min^−1^; Vt may be decreased stepwise by 0.5 ml/kg of PBW if the plateau pressure is >30 cmH_2_O).

We will set the sweeping gas flow of ECMO, targeting a PaCO_2_ range of 35–45 mmHg, and the FiO_2_ value of the ventilator may be increased if PaO_2_ in the right upper limb decreases to <60 mmHg during the course of Vt and PEEP adjustments.

Furthermore, a hybrid veno-arterio-venous ECMO mode will be considered if differential oxygenation happens (18). Differential oxygenation refers to the lower body being perfused by ECMO-oxygenated blood while the upper body (coronaries, right arm, brain) receives hypoxemic blood pumped by the native heart. This pathophysiological phenomenon often results from the recovery of native left ventricular function and simultaneous severe lung failure. If optimized ventilation fails to solve this issue, it will be necessary to employ veno-arterio-venous ECMO in consideration of the potential for hypoxic damage to the brain and heart (19, 20).

**Figure 2:**
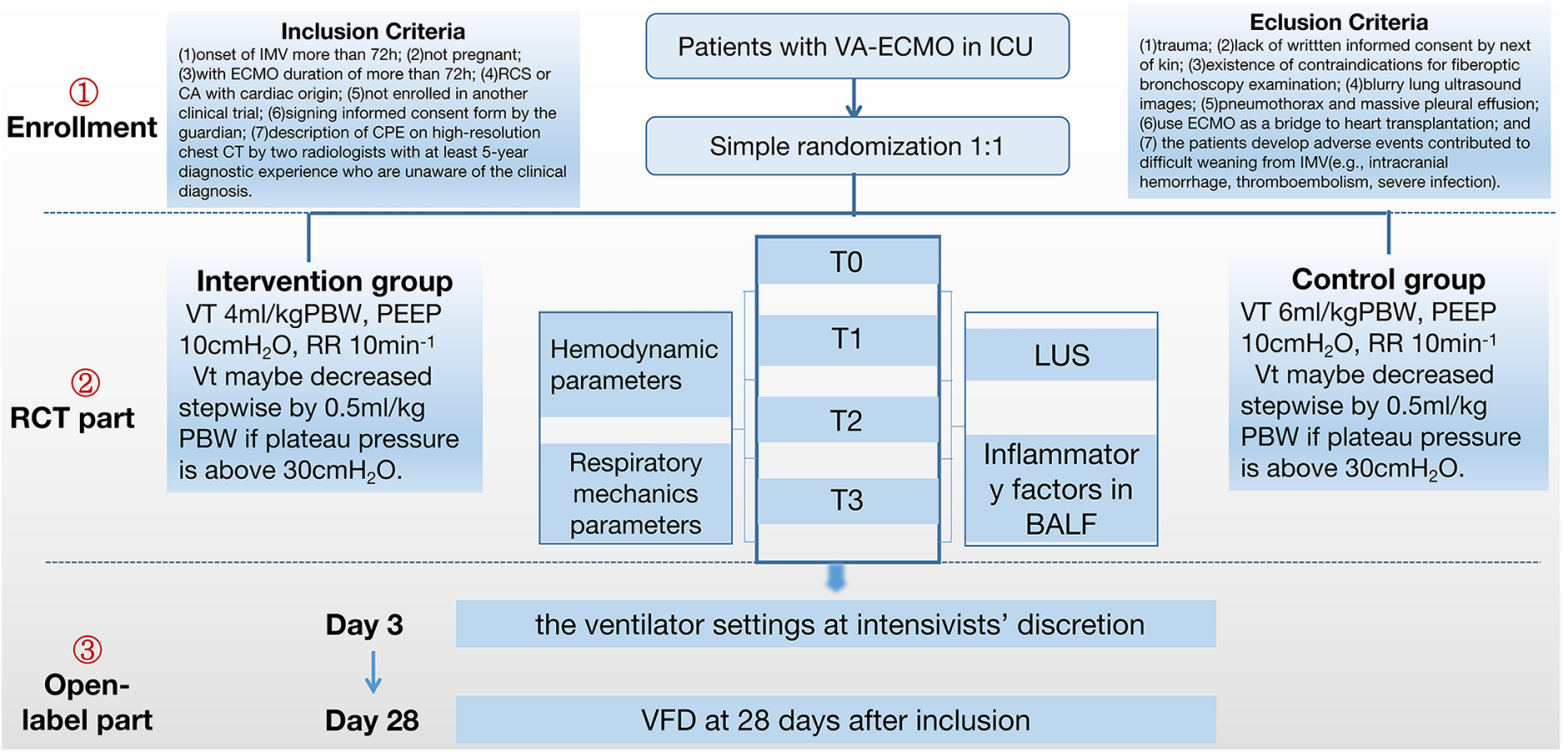
Flowchart of the study design. ECMO, extracorporeal membrane oxygenation; VT, tidal volume; PBW, predicted body weight; CA, cardiac arrest; PEEP, positive end-expiratory pressure; CPE, cardiogenic pulmonary edema; ICU, intensive Care Unit; LUS, lung ultrasound score; VFD, ventilator-free days.

At the moment of enrollment (T0), investigators will take charge of assessing and collecting pre-defined respiratory mechanics parameters; IL-6, IL-8, and MCP-1 levels; LUS; and other trial data as baseline information. The same procedure will be performed repeatedly at 24, 48, and 72 h (T1, T2, and T3, respectively) after ECMO initiation (Figure 2). After 72 h, the mechanical ventilation strategy in both groups will be changed at the intensivists' discretion.

Patients in both groups will receive deep sedation (i.e., targeting a Richmond Agitation Sedation Scale score of −3 or −4 points) during the intervention period. Although deep sedation will be adopted, it is still recommended to administer a neuromuscular blocking agent specific to the situation of patient–ventilator asynchrony.

In case adverse events occur due to the intervention, patients will be managed by the attending physicians at their discretion but still remain in the allocation group to support later data analysis.

### 2.7. Data collection and management

The total duration of ECMO treatment, total time (in days) on IMV, ICU length of stay, analgesic/sedation dosage, all-cause hospital mortality, levels of inflammatory factors in BALF, LUS, amounts of fluid infusion, and total hospitalization cost will be calculated using patient medical records (Table 6).

**Table 6:** Scheduled events and timeline for the ultra-ECMO trial.

		Study period					
Timepoint		Eligibility/Allocation	Post-Allocation			Close-out	
		T0 ECMO initiation	T1 24 h after ECMO	T2 48 h after ECMO	T3 72 h after ECMO	T4 28 days after ECMO	Tx ICU discharge day
Enrollment	Eligibility check	×					
	Informal consent	×					
	Allocation	×					
Intervention	Ultra-protective or protective VT group	×					
Assessments	Demorgraphic data	×					
	Comorbidity	×					
	Clinical characteristics	×					
	Respiratory mechanics parameters	×	×	×	×		
	LUS	×	×	×	×		
	Levels of inflammatory factors in BALF	×	×	×	×		
	In-hospital mortality		×	×	×	×	×
	RASS score	↔					
	VIS	↔					
	Amounts of resuscitative fluids		×	×	×	×	×
	Sedation and NMBA dose	↔					
	ICU LOS	↔					
	Total length of ECMO weaning	↔					
	Total lenght of IMV weaning	↔					
	Total cost for hospitalization						×
	Total hospital LOS	↔					
	28-day VFD	↔					

VT, tidal volume; VFD, ventilator-free day; ICU, intensive care unit; LOS, length of stay, VIS, vasoactive inotrope score; RASS, richmond agitation sedation scal; LUS, lung ultrasound score; BALF, broncho-alveolar lavage fluid.

We will not consider missing values for primary and secondary outcomes during hospitalization. However, missing values could appear specific to patients who survive to hospital discharge. If so, we will contact patients or their next of kin at day 28 to assess the pre-defined outcomes.

Clinical data will include but are not limited to general characteristics (age, sex, weight, height), comorbidities, original disease, the Acute Physiology and Chronic Health Evaluation II score, partial pressure of arterial oxygen/inspired oxygen fraction, PEEP, VT (in ml/kg of PBW), respiratory rate (in breaths/min), DP, P_plat_, P_es_, P_tp_, P_mean_ (in cm of water), C_stat_ (in ml/cmH_2_O), R (in cmH_2_O/L/s), arterial blood PaO_2_ and PaCO_2_ values (in mmHg), and mechanical power (in J/min).

We will also monitor the clinical conditions as well as the ECMO running status and collect any records.

Delegated team members will take charge of inputting data in the electronic case report form, and experienced clinical research associates will take charge of the data monitoring. Team members will store research data under each participant's study identification code, which will include the first letter of the first and family names of the participant, together with their inclusion number. Only the research team will have the right to possess the key to the identification code list during the study. Detailed information regarding the identification of patients will not be available in any resulting publications.

### 2.8. Sample size calculation

PASS 11 was used for sample size calculation, where a sample size of 292 patients per group would achieve 80% power to detect a difference between the two groups, providing the means were 7.1 and 9.2 with group standard deviations of 8.8 and 9.3 and with a significance level α of 0.05 using a two-sided two-sample *t*-test. Accounting for an anticipated dropout rate of 5%, 614 patients were required for the study.

### 2.9. Statistical methods

#### 2.9.1. Descriptive analysis

We will describe the demographic and clinical characteristics and compare them between the groups. All data will be given in the form of mean ± standard deviation values or medians with interquartile ranges specific to continuous variables or in the form of percentages specific to categorical variables. Student's *t* test and the Mann–Whitney *U* test will be used for comparing quantitative characteristics specific to normally and non-normally distributed variables, respectively. The *χ*^2^ test or Fisher's exact test will be used for comparing qualitative characteristics.

#### 2.9.2. Primary outcome analysis

The primary outcome is the 28-day VFD number, which will be determined by the intention-to-treat analysis (ITT) (21), regardless of whether the allocated ventilation strategy was effectively applied or not. The primary outcome will be reported in each group in the form of means with standard deviation values or medians with interquartile ranges. Student's *t* test or the Mann–Whitney *U* test will support related comparisons of the 2 groups while considering their distribution characteristics.

#### 2.9.3. Secondary outcomes analysis

The intention-to-treat analysis will help in assessing the results of the secondary outcomes. Qualitative secondary outcomes, such as the in-hospital mortality rate in each group, will be reported, and the *χ*^2^ test or Fisher's exact test will be used for the comparison. Quantitative secondary outcomes, such as LUS, will be described in each group in the form of medians with standard deviation values or medians with interquartile ranges, considering the distribution shape, and Student's *t* test or the Mann–Whitney *U* test will support related comparisons between the 2 groups. Stata version 16 (StataCorp LLC, College Station, TX, USA) will assist in carrying out all analyses. A bilateral *p*-value of <0.5 will be used to indicate statistical significance.

Performing an interim analysis will be mandatory in order to check whether there are any inappropriate operations related to data collection or management.

## 3. Discussion

In the current guidelines for ARDS, a protective ventilation strategy of limiting VT to 4–6 ml/kg of PBW is recommended, and this approach can reduce the mortality by up to 9% as proved in a randomized clinical trial (22).

Reduced VT is the mainstay of ventilatory management in ARDS, where reductions in driving pressures have been linked to a lower risk of death (23). Several authors have proposed the use of ultra-low tidal volume strategies with VTs of 4 ml/kg of PBW, which can be trialed in patients suffering from ARDS with VV-ECMO or ECCO2R-ECMO (8, 19). Tidal hyperinflation and cyclic recruitment/de-recruitment are important mechanisms leading to ventilator-induced lung injury in patients with ARDS. The mechanical ventilation strategy of ultra-protective VT or protective VT could decrease the transpulmonary driving pressure, stress, and driving pressure, thus alleviating biotrauma, volutrauma, or shearing injury. In past decades, protective mechanical ventilation strategies were extended to patients without ARDS. Fuiter et al. reported that the use of a lung-protective ventilation strategy (6.4 ± 0.8 ml/kg of PBW) in patients with intermediate and high risk levels undergoing major abdominal surgery led to enhanced clinical outcomes as well as less health care use when compared to non-protective mechanical ventilation (11.1 ± 1.1 ml/kg of PBW) (24). Neto et al. also found that ventilation with low VT (<7 ml/kg of PBW vs. ≥7 ml/kg of PBW) led to a lower risk of developing pulmonary complications in patients without ARDS (25). However, similar recommendations are unavailable in patients with refractory cardiogenic shock or CA receiving IMV, and the optimal ventilation strategy in ECMO patients is insufficiently defined (26).

Prior investigation has found that mechanical ventilation with lower VTs (odds ratio, 1.61; 95% confidence interval 1.13–2.28 per a 1-ml/kg of PBW decrease in VT; *p* = 0.008) following OHCA is associated with better neurologic outcomes (5). However, a similar conclusion has not been drawn in in-hospital CA patients (6). In short, it isn't yet confirmed whether ultra-protective VT is also feasible in the specific subject.

In the aspect of pathophysiologic mechanism, ARDS is promoted by an aggressive inflammatory reaction when there is a pulmonary or extrapulmonary assault related to altered alveolar epithelial cells or vascular endothelial cell functions. Comparatively, CPE is a type of pure hydrostatic edema attributable to cardiovascular failure. Irrespective of the pathogenesis, patients with CPE may have similar respiratory mechanics to those found in patients with ARDS (27).

**Figure 3:**
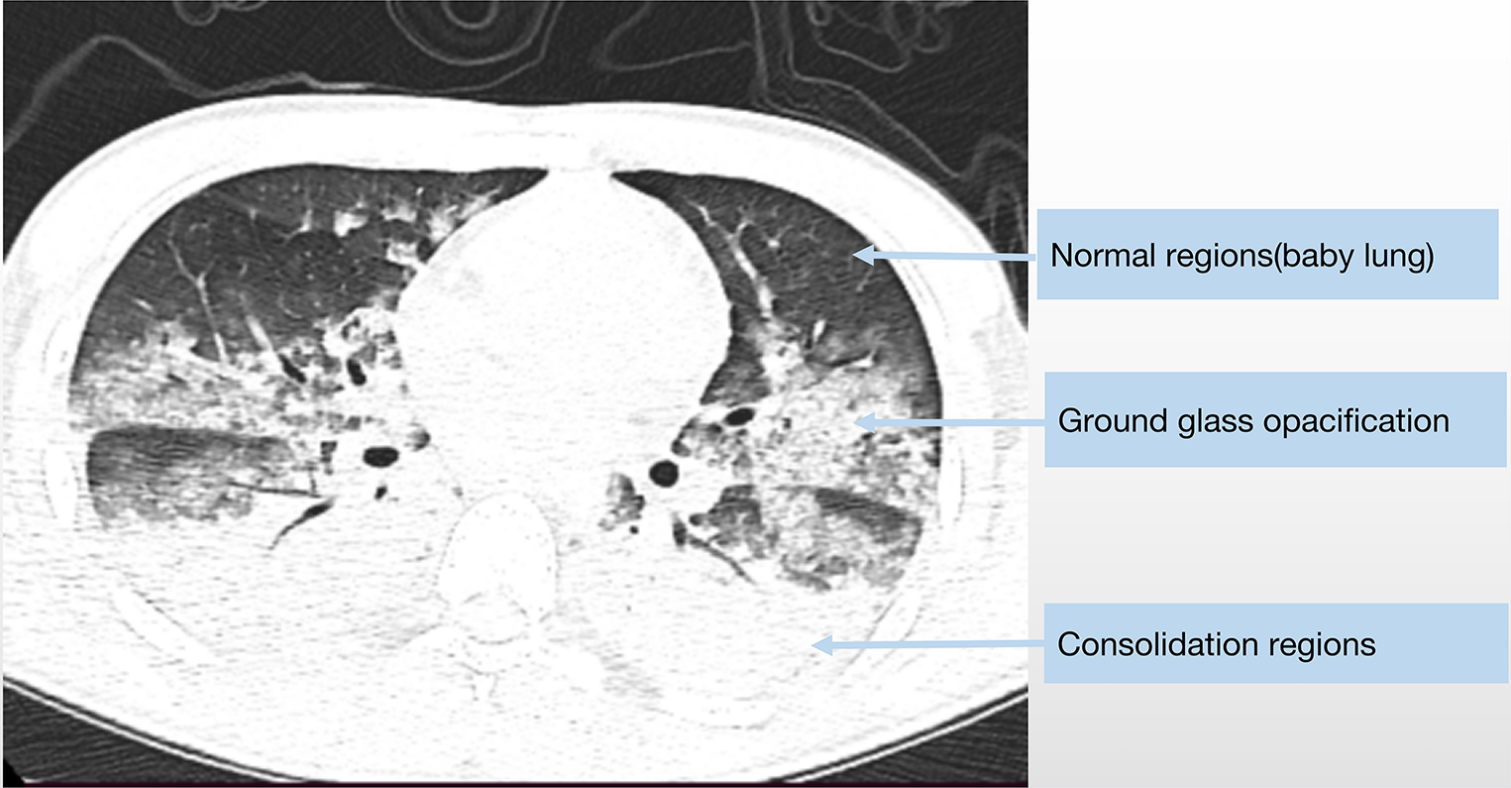
The chest CT scan of a 19-year-old male diagnosed with fulminant myocarditis show similar radiologic characteristics as classic ARDS.

In terms of radiological manifestations, the classical morphological description of an ARDS lung features an decreased amount of sufficiently inflated alveoli and increased amount of poorly inflated alveoli that move from a non-dependent region to a dependent region—namely, the normally aerated lung region, higher-density regions possessing recognizable vessels, and higher-density regions without vessels or bronchi. Analogous to ARDS patients, we also found that some patients with CPE show ARDS-mimic radiologic characteristics (namely, gravity-dependent distribution) (Figure 3).

Taken together, we reasonably hypothesize that an ultra-protective VT strategy may contribute to internal pathophysiological changes resembling those associated with an ARDS MV strategy, meanwhile be conducive to external clinical outcomes (earlier weaning of IMV, decreased LUS) in patients receiving VA-ECMO.All in all, the Ultra-ECMO trial aims to answer the question of whether early application of an ultra-protective VT strategy could facilitate weaning of IMV in patients receiving VA-ECMO.
